# Haemodynamic consequences of targeted single- and dual-site right ventricular pacing in adults with congenital heart disease undergoing surgical pulmonary valve replacement

**DOI:** 10.1093/europace/euu281

**Published:** 2014-11-04

**Authors:** Carla M. Plymen, Malcolm Finlay, Victor Tsang, Justin O'leary, Nathalie Picaut, Shay Cullen, Fiona Walker, John E Deanfield, T.Y. Hsia, Aidan P. Bolger, Pier D. Lambiase

**Affiliations:** 1 Department of Adult Congenital Heart Disease and Electrophysiology, The Heart Hospital, University College London Hospitals NHS Foundation Trust, 16-18 Westmoreland St, London W1G 8PH, UK; 2 UCL Institute of Child Health, London, UK; 3 Department of Cardiovascular Surgery, Great Ormond Street Hospital for Children, London, UK; 4 East Midlands Congenital Heart Centre, Glenfield Hospital, Leicester, UK

**Keywords:** Congenital heart disease, Resynchronization, Right ventricle, Electroanatomic map

## Abstract

**Aims:**

The purpose of this study was to create an epicardial electroanatomic map of the right ventricle (RV) and then apply post-operative-targeted single- and dual-site RV temporary pacing with measurement of haemodynamic parameters. Cardiac resynchronization therapy is an established treatment for symptomatic left ventricular (LV) dysfunction. In congenital heart disease, RV dysfunction is a common cause of morbidity—little is known regarding the potential benefits of CRT in this setting.

**Methods and results:**

Sixteen adults (age = 32 ± 8 years; 6 M, 10 F) with right bundle branch block (RBBB) and repaired tetralogy of Fallot (*n* = 8) or corrected congenital pulmonary stenosis (*n* = 8) undergoing surgical pulmonary valve replacement (PVR) for pulmonary regurgitation underwent epicardial RV mapping and haemodynamic assessment of random pacing configurations including the site of latest RV activation. The pre-operative pulmonary regurgitant fraction was 49 ± 10%; mean LV end-diastolic volume (EDV) 85 ± 19 mL/min/m^2^ and RVEDV 183 ± 89 mL/min/m^2^ on cardiac magnetic resonance imaging. The mean pre-operative QRS duration is 136 ± 26 ms. The commonest site of latest activation was the RV free wall and DDD pacing here alone or combined with RV apical pacing resulted in significant increases in cardiac output (CO) vs. AAI pacing (*P* < 0.01 all measures). DDDRV alternative site pacing significantly improved CO by 16% vs. AAI (*P* = 0.018), and 8.5% vs. DDDRV apical pacing (*P* = 0.02).

**Conclusion:**

Single-site RV pacing targeted to the region of latest activation in patients with RBBB undergoing PVR induces acute improvements in haemodynamics and supports the concept of ‘RV CRT’. Targeted pacing in such patients has therapeutic potential both post-operatively and in the long term.

**What's new?**

- Generation of an epicardial map of the electrical activation system of the right ventricle (RV) in the complex patient cohort.
- Finding that targeted single-site RV pacing is superior to apical- and dual-site RV pacing in this cohort.
- Implications for long-term bradyarrhythmia/cardiac resynchronization therapy therapy in these cohorts.

## Introduction

Adults with tetralogy of Fallot (ToF) and significant congenital pulmonary stenosis (PS) often have a right bundle branch block (RBBB) pattern on the surface electrocardiogram (ECG) resulting from earlier corrective surgery.^1^ The QRS duration frequently increases during follow-up in association with progressive pulmonary regurgitation (PR) and right ventricular (RV) dilatation.^2^ In such adults, the QRS duration has been linked with increased risk of malignant arrhythmias and sudden cardiac death, as have other depolarization and repolarization abnormalities on the surface ECG.^3,4^ Furthermore, long-term problems leading to RV dysfunction also contribute to morbidity and early mortality in this group.^5^

Due to the haemodynamic consequences of progressive PR, these patients often require re-operation in their 20′s or 30′s to implant a competent pulmonary valve. This can cause acute RV dysfunction leading to increased morbidity and mortality in the short term: studies have confirmed that across all patient groups with congenital heart disease, including those with ToF, surgery is linked to a decrease in ventricular contractile function, most marked on the second post-operative day.^6^ A sub-group of patients with ToF have a significantly poorer post-operative course, due to a restrictive cardiac physiology, requiring longer intensive care recovery with prolonged inotropic support.^7^

These patients with RBBB might therefore benefit from targeted pacing resynchronization, in both the acute and longer-term setting, provided that optimal sites within the RV can be identified. Since the outflow tract and free wall of the RV show latest activation in patients with repaired ToF,^8^ these sites theoretically constitute targets for lead placement when delivering cardiac resynchronization therapy (CRT). However, this has not been formally evaluated. Preliminary evidence has raised the tantalizing prospect of using ‘sub-pulmonary RV CRT’ to improve RV performance and clinical endpoints in outpatient populations.^9^ This is likely to be of particular importance in the early post-operative period when the RV demand increases under haemodynamic stress. Furthermore, optimization of atrioventricular delays also appears to be important.^10^ At present, relatively little is known about the electrical activation sequence, underlying mechanical dyssynchrony and subsequent potential benefits of CRT in this cohort.

This study therefore sought to define the electrical activation pattern of the RV surface in patients undergoing surgical pulmonary valve replacement (PVR) using intraoperative mapping techniques and further, to assess the haemodynamic consequences of targeted epicardial pacing at the site of latest electrical activation.

## Methods

Following local ethical approval, all adults with repaired ToF or early surgical intervention of congenital PS referred for surgical PVR during March 2010 to June 2011 were approached for inclusion into this study. All had significant PR, as defined by moderate-to-severe PR on cardiac magnetic resonance imaging (MRI, regurgitant fraction >25%) with symptoms, severe RV dysfunction or dilatation, and/or impaired exercise capacity.

Participants underwent surgical PVR at our institution (The Heart Hospital, London) done by one of two experienced surgeons (V.T., T.Y.H.). The surgical procedure has been described previously.^11^

### Electroanatomic mapping

Electroanatomic mapping was undertaken following completion of the surgical procedure, while the heart was still exposed. All patients had been rewarmed and were off cardiopulmonary bypass. All were haemodynamically stable, in sinus rhythm, and remained under general anaesthesia.

**Figure 1:**
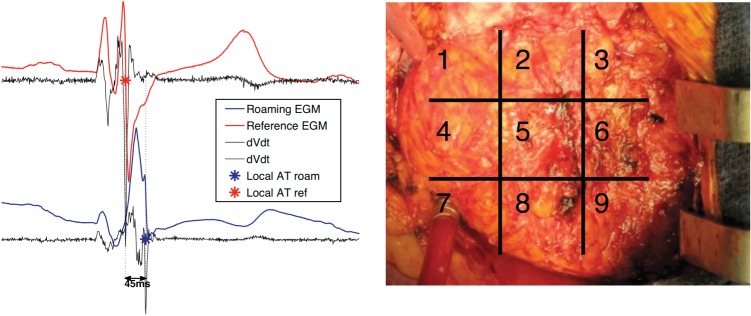
Pictorial representation of the exposed RV surface (right) with nine preselected areas to guide surgical placement of the epicardial electrode. The left panel shows a representation of the roaming and reference EGM with determination of activation of onset.

A unipolar electrode (Convenience 6494, Medtronic)was placed at the anatomical RV apex with the metal sternal retractor used as a unipolar reference. A second, roving, unipolar electrode was sequentially placed at preselected locations on the epicardial surface of the RV as determined by a nine-point grid system where the RV apex was Point 9. Unipolar electrograms (EGM) were recorded at 1000 Hz (LabSystem™ PRO EP Recording System, Bard Clearsign™, Bard, Inc.). The fastest downstroke of the unipolar electrogram (d*V*/d*t* min) was taken as the local activation time point and the activation times of the roving electrode were taken relative to the local activation time at the apex (*Figure 1*). Unipolar pacing electrodes were then fixed to the RV apex and at the latest activation site determined by electrophysiological mapping. This site was termed the ‘alternative site’ for RV pacing.

Two further atrial leads were placed as per standard procedure prior to closure of the midline sternotomy in a usual fashion.

### Acute haemodynamic study

Baseline and study measurements of cardiac parameters were derived using the Edwards Flotrac System. This device uses continuous arterial waveform analysis to provide measures of cardiac output (CO), cardiac index (CI), and stroke volume (SV) every 20 s. It is a validated alternative to invasive haemodynamic monitoring. The Flotrac system was set up preoperatively via a peripherally placed (radial) arterial line in all patients and calibrated as per the manufacturer's guidelines.

Once a patient was clinically stable on the intensive care unit (normally within 3 h of undergoing electroanatomic mapping) and while still fully sedated, pacing was undertaken. A patient return electrode was attached to the right lateral chest wall and connected directly to both ports of the external pacing device (SJM 3085 dual chamber temporary pacemaker). Both atrial and ventricular epicardial pacing wires were connected as necessary so as to generate dual chamber sensed, paced and response (DDD) RVA (apical), DDD RValt (alternate site), or DDD BiRV(apex + alternate site) pacing. Pacing was performed in all three modes at two different atrioventricular delays (intrinsic PR −20 ms; intrinsic PR −40 ms) at 10% above the patients' intrinsic heart rate. Each patient also underwent atrial sensed, paced and inhibited (AAI) pacing at this same rate, to serve as a baseline with which to compare all other modalities. Each pacing cycle was programmed for 1min to achieve the steady state, and was followed by a return to intrinsic rhythm (no pacing) between bursts lasting 2 min. Pacing order was determined using an online random sequence generator and all modes repeated three times to take into account normal physiological changes that might occur during the study period. A total of 21 random pacing cycles were therefore delivered. Results for each pacing mode are presented as an average of the three pacing cycles for that mode.

Electrocardiogram recordings were taken at each pacing mode for later analysis. These were scanned for online analysis using the CardioCalipers program (Version 3.3 for Windows, Iconico, www.iconico.com). QRS durations in each mode were averaged for each ECG following analysis of all leads, and defined as the first positive/negative deflection to the last sharp positive/negative deflection across the isoelectric line.

### Statistical analysis

Data are presented as mean ± standard deviation or median(range) as necessary. Student's paired or unpaired *t*-test or repeated-measures analysis of variance (ANOVA) were used to analyse the data where appropriate. Repeated-measures ANOVA was used to compare haemodynamic and QRS parameters in all pacing modes vs. AAI pacing were followed by Fisher's PLSD *post-hoc* analysis; all *t*-tests with Bonferroni's correction as necessary. For all repeated–measures, mean values were calculated prior to statistical analysis. A *P* value of <0.05 was considered significant. Statistical analysis was performed using StatView statistical software (SAS Institute, Inc.) and all graphs produced using GraphPad Prism (version 5.00 for Windows, GraphPad Software).

## Results

### Patient characteristics

Sixteen adults were included in this study, 50% (*n* = 8) had ToF and 50% had PS; 63% (*n* = 10) were females. All had significant PR with a mean CMR regurgitant fraction of 47 ± 10%. All were in sinus rhythm with RBBB pattern on surface ECG. Clinical characteristics of the study cohort are shown in *Table 1*. Mean age was 32 ± 11 years; mean pre-procedure QRS duration 136 ± 26 ms, mean pre-operative RV end diastolic volume (RVEDV) 183 ± 76 mL/m^2^. Ten (63%) of those included had undergone previous transannular patch (TAP) repair, seven at the time of original ToF repair.

**Table 1:** Clinical characteristics of the study cohort

	Total	ToF	Congenital PS	*P* value
Number, *n* (%)	16	8 (50)	8 (50)	−/−
Male/female, *n*	6/10	3/5	3/5	−/−
Age at PVR (year)	32 ± 11	30 ± 8	36 ± 14	0.34
Age at original repair (year)	3.3 ± 2.3	4 ± 1	2 ± 3	0.14
TAP, *n* (%)	10 (63)	7	3	−/−
Pre-procedure QRS duration (ms)	136 ± 26	137 ± 26	120 ± 4	0.20
Pre-procedure PR interval (ms)	167 ± 20	168 ± 22	166 ± 20	0.98
RVEDV (mL/min/m^2^)	183 ± 76	166 ± 40	205 ± 81	0.26
RVESV(mL/min/m^2^)	89 ± 47	80 ± 30	103 ± 65	0.43
RVEF (%)	51 ± 8	53 ± 7	49 ± 10	0.40
LVEF (%)	57 ± 10	53 ± 12	60 ± 9	0.27
PR RF (%)	47 ± 10	45 ± 10	50 ± 10	0.28

No factor was significantly different between the two anatomic substrates.

### Electroanatomic mapping

Mapping was undertaken without adverse incident in all patients. In two participants (both with ToF), electrogram analysis was not possible into two RV anterior wall segments (due to significant fibrosis causing prolonged fractionated signals).

**Figure 2:**
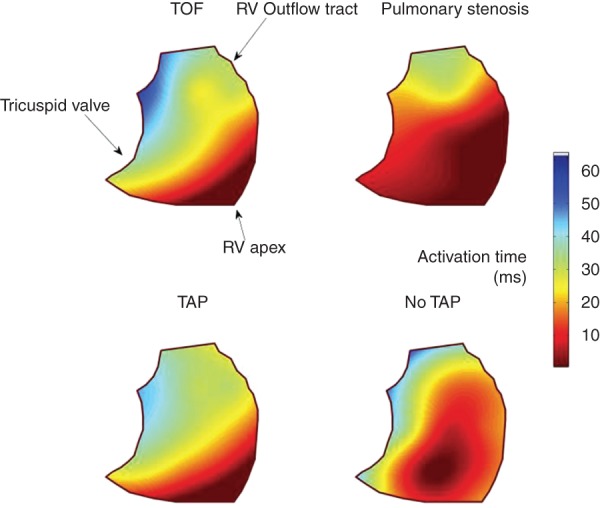
Simple colour map representation of average activation patterns across the RV in both ToF and congenital PS, with and without TAP repair.

A highly significant difference in electrical activation times across the RV was found in the whole group (*P* = 0.0013). *Post-hoc* analysis identified the RV free wall as the area most likely to be subject to delay, and electrical activation here occurred, on average, 37 ± 33 ms after the earliest activation time. The RV outflow tract was also subject to activation delay; on average activation commenced 27 ± 25 ms after the earliest local RV activation time. *Figure 2* shows a simple isochronal map representation of activation across the RV in both ToF and congenital PS, with and without TAP repair.

*Table 2* presents the activation delays at both these locations in the cohort as a whole, and per diagnosis. Interestingly, those with repaired ToF and those with previous TAP repair had significantly greater delay in the RVOT than those with either congenital PS or no TAP repair (*P* = 0.01; *P* = 0.02; *Table 2*, *Figure 2*). Those with TAP repair had the greatest activation delay in the RVOT, though this did not reach statistical significance.

**Table 2:** Activation delay compared with earliest activation time (Time 0)

	Free wall	RVOT	*P* value
Total	37 ± 33	27 ± 25	−/−
ToF	48 ± 31	41 ± 17*	0.5
Congenital PS	23 ± 32	13 ± 25*	0.4
TAP repair	37 ± 23	39 ± 16^†^	0.8
No TAP repair	25 ± 37	6 ± 25^†^	0.2

All times are in ms.

*P = 0.02 when comparing activation times at the RVOT in those with ToF vs. congenital PS anatomy.

^†^*P* = 0.01 when comparing activation times at the RVOT between those with TAP and no TAP repair.

### Acute post-operative pacing

All recruited patients were paced, and there were no pacing-related adverse events. Furthermore, all patients were haemodynamically stable throughout recovery and none required formal temporary pacing during this time. No patient included in this study required inotropic support and all remained haemodynamically stable during the study.

All modes of pacing, including AAI pacing, generated increases in CO significantly above baseline (*Table 3*). Mean arterial pressure did not change significantly with any mode of pacing (*P* = 0.49). DDDRValtpacing at the site of latest RV activation with AV delay minus 20 ms of intrinsic was associated with the greatest overall increase in CO including significant improvements over AAI pacing alone(*Table 3*), and this significance was maintained when directly compared with DDDRV apical pacing at the same AV delay: CO (5.8 ± 1.3 to 6.4 ± 1.7; *P* = 0.018). DDDRValt pacing increased CO by 16% greater than achieved by AAI pacing (*P* = 0.018) and by 8.5% above DDDRV apical pacing (*P* = 0.02).

**Table 3:** Haemodynamic and pacing parameters measured with each pacing mode

	Baseline (intrinsic)	AAI	DDD RVA −20 ms	DDD RVA −40 ms	DDD RValt −20 ms	DDD RValt −40 ms	DDD BiRV −20 ms	DDD BiRV −40 ms	*P* value (ANOVA)
CO (L/min)	4.7 ± 1.2^†^	5.5 ± 0.9	5.8 ± 1.3*	5.9 ± 1.3	6.4 ± 1.7*	6.1 ± 1.4	5.9 ± 1.5	5.9 ± 1.3	0.003
CI (L/min/m^2^)	2.7 ± 0.7^†^	3.2 ± 0.5	3.4 ± 0.8	3.5 ± 0.9	3.7 ± 1	3.6 ± 0.9	3.5 ± 0.9	3.5 ± 0.8	0.004
SV (mL)	58 ± 16^†^	61 ± 10	63 ± 13	64 ± 13	68 ± 17	66 ± 14	65 ± 15	65 ± 13	0.01
MAP (mmHg)	70 ± 11^†^	74 ± 10	72 ± 10	73 ± 9	72 ± 10	73 ± 10	74 ± 9	74 ± 10	0.49
QRS (ms)	136 ± 26	116 ± 18	138 ± 20	143 ± 22	127 ± 20	132 ± 25	132 ± 25	129 ± 29	0.16

All forms of pacing, including AAI pacing, generated increases of CO significantly above baseline; however, DDD RV alternate site pacing was found to generate the greatest increase in all measured parameters. There was no significant difference in MAP or QRS duration in the cohort with pacing.

^†^Not included in repeated-measures ANOVA.

*P = 0.018 when comparing CO DDD RValt with AAI pacing.

### QRS duration

The mean QRS duration for the whole-cohort pre-procedure was 136 ± 26 ms. Although a visible trend is seen, there was no significant difference in QRS duration between those with a diagnosis to ToF or congenital PS (137 ± 27 vs. 120 ± 26; *P* = 0.20) or those with and without a history of TAP repair (134 ± 23 vs. 116 ± 8; *P* = 0.16). The QRS duration did not correlate with the CO generated by any form of pacing.

**Figure 3:**
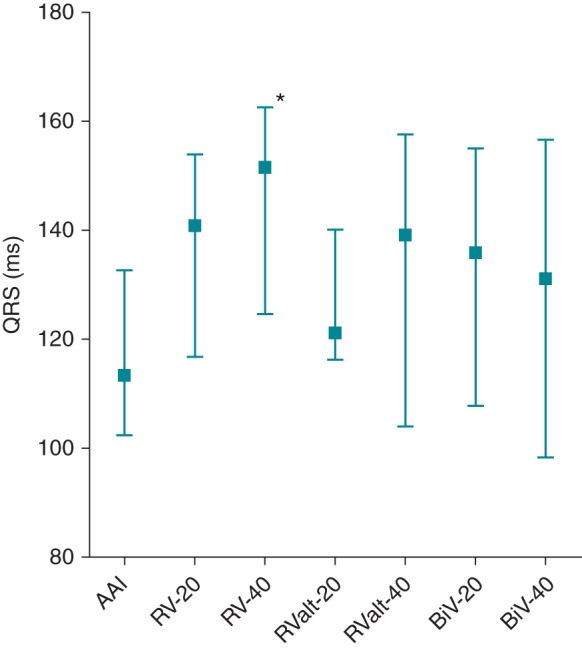
Graph showing absolute change (±SD) in QRS duration with various pacing modalities. Although none was significantly better than AAI pacing, RV apical pacing alone, with either −20 ms or −40 ms AV delay, was associated with the longest QRS duration generated.

Both forms of RV apical pacing were associated with a significantly wider QRS duration than occurred during AAI pacing, though there was no significant difference in QRS duration when comparing the alternate pacing sites (RVA to RValt −20 ms, *P* = 0.11; RVA to RValt −40 ms, *P* = 0.07). *Figure 3* and *Table 3* present the mean data for each pacing programme. Full QRS data on two patients (one ToF, one PS) was not available.

## Discussion

This prospective study examined targeted single- and dual-site pacing in the RV of adults with corrected tetralogy of Fallot or PS. Electroanatomic mapping confirmed that while there is no uniform pattern to activation of the RV, the latest area of electrical activation in these adults is often the RV free wall; however, the RVOT is subject to the greatest variation in activation, being latest in those with TAP repair and/or ToF. We confirm that targeted (alternate) single site RV pacing significantly improves cardiac haemodynamics acutely, more so than RV apical pacing or dual site RV pacing. Right ventricular apical pacing alone either exerted a minimal or detrimental effect on haemodynamics.

### Electrical activation of the right ventricle

Analagous to LBBB, RBBB is a complex electrical disease. The findings of a lack of uniformity to activation across the cohort are in keeping with the knowledge that right bundle branch block can occur at different levels, proximal, distal, and terminal, depending on the location and extent of the original (surgical) insult.^1^ Furthermore, although all these levels of block manifest different electrical activation sequences on electrophysiological mapping, they are represented by a similar RBBB pattern on the surface ECG. Therefore, when directly comparing those patients who had a transannular patch repair with those who had not, it is perhaps unsurprising to note that the former had significant delays in electrical activation compared with the latter. Thus, although both the RV free wall and RV outflow tract had similar levels of activation delay in the whole cohort, the RV outflow tract was subject to the greatest variation, depending on the anatomy and nature of the original repair.

These results correspond to a high density epi- and endocardial mapping study of Fallot's patients which demonstrated that the RVOT and infundibular area was the latest site of activation in 73% of the 15 cases studied—indeed the degree of delay between the apex and the RVOT was equivalent to our findings of ∼50 ms when the RVOT was the latest activation region.^12^ Furthermore, significant LV epicardial activation delays were also identified, indicating that RBBB in ToF is linked to LV electrical dyssynchrony. Importantly, this latter study did not examine the effects of pacing on haemodynamics. However, in a limited combined body surface mapping and biventricular pacing study of ToF patients, the infundibulum was found to be the latest site of activation and BiV (i.e.; RV–LV) pacing improved exercise capacity and dyssynchrony parameters.^13^ Interestingly, targeted latest site RV pacing was not performed, only conventional RV apical pacing and BiV stimulation.

We did not find the RV apex was subject to significant delay, in keeping with previously published studies.^8,12^ Furthermore, the degree of RV volume overload and PR was similar in the whole cohort, as was LV function (*Table 1*), suggesting that haemodynamic factors were not related to the electrophysiological findings.

### Resynchronization pacing

Regardless of the underlying cause, the level of functional block and its consequent effect on the local mechanical response are of paramount importance when considering the delivery, and thus efficacy, of CRT.

It is interesting to note that the haemodynamic parameters in this study improved with all forms of pacing, including AAI and RV apical pacing, from baseline values. This acute beneficial effect of pacing may well be related to rate increase or AV shortening, facilitating optimal LV diastolic filling. However, further study allowing for intrinsic sinus rate with VDD pacing would be required to investigate this further, perhaps utilizing 3D TOE and epicardial high density electrode mapping to assess both activation and haemodynamic effects.

This study does demonstrate that targeted, alternate site, RV pacing leads to superior improvements in CO when compared with AAI pacing alone, or DDD RV apical pacing, in adults late after repair of ToF or congenital PS. Interestingly, the combination of targeted alternate site RV pacing and RV apical pacing (DDD BiRV) does not lead to a greater improvement in haemodynamics, and indeed also appears inferior to alternate site RV pacing alone.

This is the first study in this population to directly compare DDD RV apical pacing and thet best alternate site RV pacing. Although it supports the concept of ‘sub-pulmonary RV CRT’ in this cohort, long-term effects in terms of iatrogenic dyssynchrony need to be established. Thambo *et al*.^14^ reported that such therapy was associated with marked dyssynchrony of both the LV and the RV in a small cohort, though RV pacing sites were not directly targeted and dyssynchrony was most marked with RV apical pacing. The fact that considerable epicardial conduction delays also affect the LV in ToF has important implications for lead placement.

Although RV disease is predominant in this congenital population, it does not occur in isolation and the possibility of iatrogenic dyssynchrony in the long-term should be considered in the sub-aortic LV, especially if the systolic function of this chamber is already impaired. Abd El Rahman *et al.*^5^ describe delayed activation of the LV in an around half of the patients with repaired ToF and RBBB.^5^ The pattern differed from that seen in patients with dilated cardiomyopathy and RBBB, affecting the ventricular septum and not the lateral LV wall. In a retrospective analysis of 75 patients with repaired ToF, Tzemos *et al*.^15^ noted a significant association between QRS duration and adverse LV volume, activation delay and septal strain, and recently, the Toronto group published their observations of significant delays in LV activation in their study group of adults with repaired ToF. It is already well established that RV apical pacing, both acutely and in the long term in the general population, is associated with LV dyssynchrony and functional impairment. Our current findings indicate that such dyssynchrony as may be induced by additional RV apical pacing (i.e. with dual site RV pacing) may be sufficient to abrogate the benefits of targeted single-site resynchronization.

The acute nature of this current study needs to be considered also: The phenomenon of ventricular interdependence explains how early surgical intervention on the RV and the subsequent effects of progressive haemodynamic lesions may negatively impact the LV function by electromechanical uncoupling.^16^ A recent study looked at the degree of excursion of the intraventricular septum, the main mediator of this phenomenon, in patients with repaired ToF. The group determined that in those with abnormal excursion there was a reduction in global and septal LV systolic function.^17^ A study by Tobler *et al.* determined that LV function improved in adults with ToF several years following surgical PVR, supporting the recovery of adverse electromechanical interactions between the two chambers.^18^ This finding has been documented elsewhere also;^19^ however in both studies patients improved when there was already LV functional impairment. Long-term RV resynchronization by pacing would be hoped to have a similar outcome on LV function but this remains to be shown.

In terms of ECG findings, the cohort was too small to determine any significant difference between the respective groups although there was a trend to QRS prolongation between different anatomic substrates (ToF vs. congenital PS) and previous surgical intervention (TAP vs. no TAP), as would be expected.

We find that RV apical pacing promotes lengthening of the QRS width, as others have demonstrated previously,^10^ although this did not reach statistical significance in our study. Stephenson *et al.*^10^ document subsequent shortening of the QRS duration with AV optimization, and it may be that in our cohort of patients, many of whom had QRS intervals within the normal range (RV dyssynchrony was not a prerequisite for entry), this was sufficient to facilitate electrical optimization such that targeted pacing in addition to AV optimization did not result in additional QRS shortening. Right ventricular apical pacing did significantly prolong QRS duration vs. AAI pacing, but alternate site RV pacing did not, indicating that it was inducing less electrical dyssynchrony, reflecting the improved haemodynamic response to this mode.

### Clinical implications

In this study, single-site, targeted RV pacing led to significant improvements in cardiac haemodynamics. In the acute setting, in those who suffer more protracted post-operative courses linked to adverse RV function, targeted resynchronization pacing could be of significant benefit in the immediate recovery period, especially since it seems clear that RV apical pacing is deleterious at worst and at best neutral in effect. In terms of routine temporary pacing wire placement, these data indicate that epicardial RV leads should be placed in the outflow tract/free wall area of the RV as opposed to the apex.

## Limitations

This was an acute study in a small population of patients and should serve as a preliminary assessment of targeted RV pacing in this cohort. Invasive assessment of RV d*P*/d*t* may have provided more specific RV data acutely. Further, calculation of invasive SVR, not undertaken in this study, would provide more information as to the mode of increase in CO without concomitant rise in arterial BP. Care must be taken, however, with regards to fluid shifts in this early post-operative phase.

Only the site of latest activation was evaluated; it was not possible to systematically test all segments and so it is possible that other segments could have resulted in greater haemodynamic changes. Although our study patients underwent early post-operative transthoracic echocardiography, it was not possible to consistently image the RV and LV in detail due to limited accurate and reproducible acoustic windows. An imaging study would require longer-term lead placement or intraoperative 3D transoesophageal echocardiography to determine the mechanical effects of pacing. Further determination of pre-operative and post-operative LV, RV, and interventricular dyssynchrony would provide crucial information on the interrelations between these two chambers in response to single-site, targeted RV pacing. Future long-term studies utilizing endocardial pacing techniques would help to facilitate this. A future study could incorporate both high-density epicardial sock mapping and intraoperative 3D TOE to fully interrogate the acute electromechanical effects of alternate site RV pacing intraoperatively. With the evolution of MRI safe pacing technology, studies utilizing MRI could be performed to investigate RV/LV remodelling with long-term alternate site pacing targeting the region of latest RV contraction analogous to the TARGET trial of CRT.^20^

Longer-term studies will be necessary to determine the impact of alternate site pacing in a population of ToF patients with RBBB and RV impairment.

There was a further limitation in the epicardial nature of our electroanatomic mapping, in that septal activation could not be assessed. This would likely change the total time taken for RV activation. Future studies incorporating endocardial mapping techniques would resolve this issue.

## Conclusion

Single-site RV pacing targeted to the region of latest intrinsic activation in patients with RBBB undergoing PVR induces acute improvements in central haemodynamics and supports the concept of sub-pulmonary ‘RV CRT’. Targeted pacing in such patients has therapeutic potential both in the post-operative and chronic settings and is superior to RV apical pacing which may be detrimental.

**Conflicts of interest:** P.D.L. receives educational grant from Boston Scientific. C.M.P. is funded by a Clinical Research Fellowship grant from the British Heart Foundation: FS 09/041/27772. M.F. is supported by the Wellcome Trust.

## Funding

The work was undertaken at UCL/UCLH which receives a proportion of its funding from the Department of Health′s NHIR Biomedical Research Centres Scheme. Funding to pay the Open Access publication charges for this article was provided by University College London.
